# Effects of Chemical Fertilization and Microbial Inoculum on *Bacillus subtilis* Colonization in Soybean and Maize Plants

**DOI:** 10.3389/fmicb.2022.901157

**Published:** 2022-07-06

**Authors:** Clara Barros Bueno, Roberta Mendes dos Santos, Fernando de Souza Buzo, Maura Santos Reis de Andrade da Silva, Everlon Cid Rigobelo

**Affiliations:** ^1^School of Agricultural and Veterinarian Sciences, São Paulo State University (UNESP), Jaboticabal, Brazil; ^2^Agricultural and Livestock Microbiology Graduation Program, São Paulo State University (UNESP), School of Agricultural and Veterinarian Sciences, Jaboticabal, São Paulo, Brazil; ^3^Department of Plant Science, Food Technology and Socioeconomics, Faculty of Engineering of Ilha Solteira/UNESP, Ilha Solteira, Brazil

**Keywords:** plant growth-promoting bacteria, inoculum concentration, fertilization doses, *Glycine max*, *Zea mays*

## Abstract

Plant growth-promoting endophytic microorganisms in agriculture have been expanding in Brazil and are an excellent strategy to face the challenges of current agriculture, such as reducing production costs with fewer environmental impacts, without detriment to productivity. However, little is known about the factors that can affect the colonization of endophytic such as inoculant concentration and mineral fertilization. The present study aimed to evaluate the influence of these factors on soybean and maize crops and found that for soybean crops, the highest *Bacillus subtilis* concentration of 1 × 10^4^ and 1 × 10^10^ CFU ml^−1^ promoted the highest number of recovered bacteria, when there was no mineral fertilization. However, mineral fertilization limited the number of recovered bacteria, suggesting that mineral fertilization interferes with endophytic colonization. For maize crops, the highest number of recovered bacteria occurred from the concentration of 1 × 10^6^ CFU ml^−1^, not differing from the highest concentrations. A mineral fertilization dose of 25% promoted the greatest *B. subtilis* recovery compared to the other treatments. Regarding plant development, the highest microbial inoculum concentrations did not necessarily promote greater positive growth promotion effects compared to the concentration of 1 × 10^4^ CFU ml^−1^ for both crops. The results also suggest that the higher number of endophytic bacteria recovered in the plant does not necessarily affect plant growth in the same proportion. For soybean plants, there is a strong tendency that with the increase in the *B. subtilis* inoculant concentration, the need for mineral fertilization doses to achieve the same plant development is consequently increased, and inoculations with 1 × 10^5^ and 1 × 10^6^ CFU ml^−1^ with fertilization doses between 44% and 62% are the ideal combinations for greater plant development. In maize plants, the best growth promotion response (height) was obtained using inoculation concentration of 1 × 10^2^ and 1 × 10^10^ CFU ml^−1^, increasing according to the increase in fertilization doses. The findings suggest, for soybean crop, that these high inoculum concentrations required more photosynthetic metabolites from the plants and more nutrients from the soil. Thus, the need for mineral fertilization for plant growth must be increased.

## Introduction

Since the appearance of plants, microorganisms are associated with them, allowing the emergence of different types of interactions that can be beneficial, harmful or neutral (Turner et al., 2013). When the relationship is harmful to the plant, this results in a decrease in its development, while a beneficial relationship promotes improvement in its development (Downie, 2010).

Microbial communities in plants represent a potential solution to the decline in global food production. Plant growth and health are modulated by microbial communities that colonize their tissues. Although some microbes are detrimental and cause diseases, others may promote plant growth and tolerance to biotic and abiotic stresses by enhancing nutrient acquisition *via* many mechanisms (Brader et al., 2017).

The application of plant growth-promoting microorganisms (PGPs) may improve plant production under unfavorable conditions, with the potential to reduce the use of chemical fertilizers and pesticides. However, the current application of endophytes, especially in the field, faces a number of challenges. Microbial application may not completely replicate the effectiveness of chemical fertilizers (Sessitsch et al., 2019).

Many mechanisms that are related to plant growth promotion necessitate further investigation, including the steps involved in plant colonization by PGPs, plant–soil interactions of root endophytes, and microbes associated with all plant parts. Additionally, the interaction of microbes living within the same plant, how they modulate above- and belowground communities, and the involved processes all require further examination to improve the efficiency of the inoculant strain (Compant et al., 2016).

Bacteria that interact with plants are classified according to their location and can be rhizospheric, epiphytic and endophytic. Most plant-associated bacteria are derived from the soil environment (Compant et al., 2010). Microorganisms that live inside the plant are called endophytes. Endophytes consist of several microorganisms that spend either their full or partial life cycle colonizing plant tissues. Endophytic bacteria have the ability to invade the internal tissues of living plants without developing disease symptoms during their life cycle or in part of it (Hartlova et al., 2011).

The endophytic environment is more protected than the soil or rhizosphere, giving an ecological advantage to the other bacteria capable of colonizing it (Berendsen et al., 2012). However, in plant endophyte interactions, bacteria do not reside within cells and do not induce the formation of differentiated plant structures, such as nodules, in rhizobia-legume interactions (Knief et al., 2011).

Bacteria within the host find great availability of nutrients, low competition and protection against environmental stress, and in some cases, this lifestyle guarantees their dispersion by vertical transfer (Compant et al., 2021). The life of bacteria within plant tissue allows for a more intimate interaction with the host, effectively influencing the plant phenotype (Downie, 2010).

There is strong evidence that many apparently commensalistic endophytes can also promote plant growth and defense (Santoyo et al., 2016; Hossain et al., 2017), but the ecology and functions of these beneficial endophytes are poorly understood. In particular, endophytic plant growth-promoting bacteria (PGP) can promote plant growth by mechanisms that include the release of phytohormones (Patten and Glick, 2002), nitrogen fixation (Urquiaga et al., 2011; Sun et al., 2022), improved mineral acquisition (Malinowski et al., 2000; Rahman et al., 2005; Kushwaha et al., 2022), production of growth-promoting compounds (Kumar et al., 2019) and increased stress tolerance (Kohler et al., 2008; Aziz et al., 2022). However, little is known about the factors that directly or indirectly influence the colonization of endophytic bacteria in plants.

In this context, the present study verified whether two factors, bacterial inoculant concentration and chemical soil fertility, interfere with the colonization of the endophytic bacterium *B. subtilis* and, consequently, whether the number of endophytic bacteria interferes with plant growth promotion in soybean and maize crops.

For this, the development of soybean and maize plants was evaluated at different *B. subtilis* concentrations marked with the GFP at concentrations from 1 × 10^2^ to 1 × 10^10^ CFU ml^−1^ at four mineral fertilization doses of 0%, 25%, 50% and 100% according to the chemical soil analysis under greenhouse conditions.

### Objective

The aim of the present study was to verify whether the colonization of the endophytic bacterium *B. subtilis* is influenced by the bacterial inoculant concentration and by the soil fertility conditions and whether plant development is influenced by the endophytic bacterial population.

## Materials and Methods

### *Bacillus subtilis* Transformation

The method used for *B. subtilis* BS-290 transformation was described by Anagnostopoulos and Spizizen (1961), with some modifications (Rosenberg et al., 2016). As the *Escherichia coli* strain is easily transformed, this strain was also used as a positive control for transformation.

*Bacillus subtilis* and *E. coli* strains were cultivated in minimal medium that is appropriate for bacterial transformation, called SP, composed of 50% glucose, 5 mg/ml tryptophan, 22 mg/ml ammonium iron citrate, 1 M magnesium sulfate, 40% K-glutamate, 5% hydrolyzed casein and 9.215 ml/10 ml SP salts (K_2_HPO_4_ 14 g/L; KH_2_PO_4_ 6 g/L; trisodium citrate 1 g/L). This medium was used throughout the process of bacterial competence and transformation.

To facilitate the entry of DNA, *E. coli* and *B. subtilis* were inoculated in 5 ml of SP medium overnight growth at 37°C under stirring at 150 rpm. The next day, the culture was diluted at 1:50 into a new sterile SP medium and again placed in incubation at 37°C with 180 rpm under stirring. Optical density was measured in a spectrophotometer with an absorbance of 600 nm every 30 min after 2 h of incubation until a change in bacterial growth from the exponential growth phase to the stationary phase was observed, which took approximately 5 h 30 min. After this period, 400 μl of each culture was placed in Eppendorf tubes, together with 600 ng of pGLO plasmid plus fluorescent protein (GFP) and ampicillin resistance gene. Tubes were separated with and without plasmid application and incubated again for 90 min under the same temperature and stirring conditions.

At the end of this period, 100 μl of the incubated bacterial solution was plated on Luria-Broth agar medium (5 g of NaCl; 5 g of yeast extract; 10 g of tryptone and 15 g of agar, for 1 L) for bacteria without pGLO. LB culture medium containing ampicillin and arabinose was prepared for bacteria containing plasmid only. After 24 h of growth at 37°C, these bacteria were inoculated in liquid brain heart infusion (BHI) medium and incubated for another 24 h at 28°C to be later plated in BHI with arabinose and ampicillin and only with ampicillin. Bacteria submitted to the previously described competence process, but not receiving the plasmid, were plated in medium with ampicillin (100 μg ml^−1^), as well as bacteria that received the plasmid and those that were not even submitted to the starvation process.

### Experimental Conditions

In March 2020, the soybean (*G. max*) variety AS-3595 Company Agroceres was planted, and in July, the maize (*Z. mays*) variety BRS-3042—Company Embrapa—was planted; both experiments lasted 45 days.

Experiments were carried out in a greenhouse in the municipality of Jaboticabal, Sao Paulo State (21° 15′ 17” S and 48° 19′ 20” W). The greenhouse conditions were maintained at a temperature of 24 ± 2°C, watering 50 ± 2% RH, and 250 μmol light, 16:8 h L:D. According to Embrapa (2006), the region has eutrophic red latosol with clayey texture.

### Experimental Design and Pot Preparation

Both experiments with soybean and maize had the same experimental design, composed of randomized blocks and in a 10 × 4 factorial with five replicates, with 10 *B. subtilis* inoculum concentrations (control and concentrations from 1 × 10^2^ to 1 × 10^10^ CFU ml^−1^) and four fertilization doses (0%, 25%, 50% and 100%). Fertilization followed the recommendation of Malavolta (1980) for 5-L pots and soils with pH values between 6.0 and 6.5. The manganese recommendation is not present in Malavolta’s book (1980), so values from the work of Sánchez-Molina et al. (2014) were used.

The fertilizers used were urea (45% N), super simple (18% P_2_O_5_, 16% Ca, 8% S), potassium chloride (60% K_2_O), calcium carbonate (16% Ca), magnesium oxide (52% Mg), boric acid (17% B), molybdate (39% Mo), copper sulfate (25% Cu), zinc sulfate (20% Zn), and manganese sulfate (26% Mn), and calculations were performed according to the desired percentage for the four fertilization doses.

### Production of Inoculants

Inoculant with the already transformed *B. subtilis* 290 accession number in GenBank (MZ133755) was cultivated in nutrient broth media (meat extract—1 g/L; yeast extract—2 g/L; peptone—5 g/L; sodium chloride—5 g/L; final pH 6.8 ± 0.2) for concentrations from 1 × 10^2^ to 1 × 10^7^. To achieve high concentrations from 1 × 10^8^ to 1 × 10^10^ CFU ml^−1^, the bacterium *B. subtilis* was cultivated in Brain Heart Infusion broth (calf brain—200 g/L; beef heart—250 g/L; proteose peptone—10 g/L; dextrose—2 g/L; sodium chloride—5 g/L; disodium phosphate—2.5 g/L; final pH 7.4 ± 0.2), and both media were incubated in BOD at 28°C for 24 h (Lobo et al., 2019). All the concentrations were adjusted by serial dilution.

### Planting and Inoculations

Maize and soybean seeds were sown in pots with five liters of unsterilized soil previously chemically analyzed and fertilized as mentioned above. After the emergence of plants, thinning was carried out, leaving two plants per pot.

Inoculum dilutions were standardized by reading in a spectrophotometer at 630 nm (Kloepper et al., 1989), and each pot received 10 ml of inoculant, adjusting concentrations from 1 × 10^2^ to 1 × 10^10^ CFU ml^−1^. Inoculations started after 7 days of germinated seeds and occurred every 10 days, totaling four inoculations.

### Parameters Evaluated

#### Plant Height and Fresh Matter

Height was measured from the plant base (close to the ground) to the apex with the aid of a measuring tape. Shoots and roots were carefully separated, and roots were washed with the aid of a water jet and dried on absorbent paper for later fresh matter measurement on a semianalytical scale. As a consequence of the need for the previous use of parts of roots to perform *B. subtilis* reisolation, it was not possible to perform dry matter measurement, but total fresh matter measurement; otherwise, the analyses mentioned above would be unfeasible.

#### Shoot Nitrogen Concentration

The process followed the method of Vieira and Nahas (2005) previously proposed by Bremner and Mulvaney (1982). Approximately 0.1 g of vegetable sample was measured and added to the digester tube together with 7 ml of digester mixture to follow in the digester block according to the time and temperature sequence of 1 h at 100°C, 1 h at 200°C and 1 h at 300°C. Subsequently, the digested material was cooled and dissolved in 10 ml of distilled water. Distillation took place in a Kjeldahl semimicro distiller with 25 ml of NaOH (50%). The distilled material was collected in 10 ml of boric acid solution as an indicator to obtain a total volume of 20 ml. The distilled ammonia was titrated using standard 0.05 N H_2_SO_4_ solution, and titration ended when the color changed from green to light red, considering that 1.0 ml of 0.05 N sulfuric acid in the titration corresponds to 0.7 g of N in the sample.

#### Shoot Phosphorus Concentration

Approximately 0.5 g of the vegetable sample was added to the digester tube together with 5 ml of concentrated nitric acid and 1 ml of perchloric acid so that overnight resting nitro-perchloric predigestion occurred. After this period, complete digestion in a digester block was performed according to the time and temperature sequence of 1 h at 80°C, 1 h at 120°C, 1 h at 150°C and 1 h at 180°C. After washing the digested material with distilled water, 50 ml of extract was obtained. Then, reading was performed in a spectrophotometer at 470 nm using 5 ml of extract and 1 ml of reagent (mixture of equal volumes of 5% ammonium molybdate and 0.25% ammonium vanadate).

### Reisolation of Transformed Isolates

The reisolation of transformed *B. subtilis* isolates was carried out 45 days after planting. Reisolation occurred in roots, which were separated and washed in running water to remove aggregated soil and other impurities.

Surface disinfection of tissues was performed using methodology described by Santos et al. (2018). Tissues were cut, and 0.5 g was aseptically weighed and macerated with 4.5 ml of 0.1% NaCl. The dilution to reduce the inoculum concentration consisted of adding 1 ml from each tube to another with 9 ml of saline solution. Tubes with the specific dilution were submitted to heat shock treatment at a temperature of 80°C for 10 min, and then 100 μl of suspension was plated in BHI culture medium containing ampicillin. Petri dishes were placed in growth at 28°C, and colonies were counted at 24 and 48 h. The results were expressed in CFU of *B. subtilis* per gram of plant shoot.

### PCR – 16S rDNA Sequencing

To confirm the bacterial species, the bacterial strain was reisolated, and its DNA was extracted according to the instructions of the *Quick-DNA Universal Kit (Zymo Research—cat. N° D4068 e D4069)* kit. DNA amplification was performed by 16S rDNA at a final volume of 25 μl containing all reagents needed to react in μl ultrapure sterile water, 11,3; 10 millimolar primer F, 1,5; 10 millimolar primer R, 1,5; Taq green Buffer 5x, 5,0; MgCl_2_, 3,0; 10 millimolar each dNTP, 1,0; Taq DNA polymerase, 0,2; DNA mold, 1,5. Genomic DNA was extracted using the Quick-DNA Universal Kit (Zymo Research) following the manufacturer’s instructions. 16S ribosomal DNA was amplified by polymerase chain reaction (PCR) using the primers P027F (5′-GAGAGTTTGATCCTGGCTAG-3′) and 1378R (5′-CGGTGTGTACSSGGCCCGGGAACG-3′) with the amplification program: 95°C for 2 min; followed by 25 cycles of denaturation at 95°C for 30 s, annealing at 63°C for 1 min and extension at 72°C for 1 min, and a final extension at 72°C for 7 min. The PCR products were purified and sequenced in an automated DNA ABI3730 sequencer using the primers P027F and 1378R. The sequences were aligned and edited using BioEdit 7.0.5.3 software (Hall, 1999) and compared to the sequences from GenBank at the NCBI (National Center for Biotechnology Information). Phylogenetic analyses were performed using MEGA 6.0 software (Tamura et al., 2013).

### Data Analysis

The results were submitted to analysis of variance (ANOVA), and when significance was verified by the F test, means were compared by the Tukey test (*p* < 0.05). To evaluate the effect of fertilization doses and *B. subtilis* inoculant concentrations, regression analyses were performed (*p* ≤ 0.05). All procedures were performed using SISVAR 5.0 statistical software (Ferreira, 2011).

## Results

### Transformation

*Bacillus subtilis* BS-290 colonies did not emit fluorescence in the presence of arabinose, as occurred with *E. coli*. The bacterium *E. coli* was used as a positive control for transformation. However, it was observed that the transformation was successful, as the ampicillin resistance gene was expressed, allowing the bacterium to grow in culture medium in the presence of ampicillin, whereas the same untransformed isolate did not grow in the presence of ampicillin. Therefore, the confirmation of the presence of reisolated and endophytic bacteria previously inoculated in plant roots was due to the growth of isolates in medium with the presence of ampicillin at the aforementioned concentration. The reisolated *B. subtilis* strain was identified through automatic sequencing to prove its identity (Supplementary Figure S1).

### Soybean Crop

**Figure 1:**
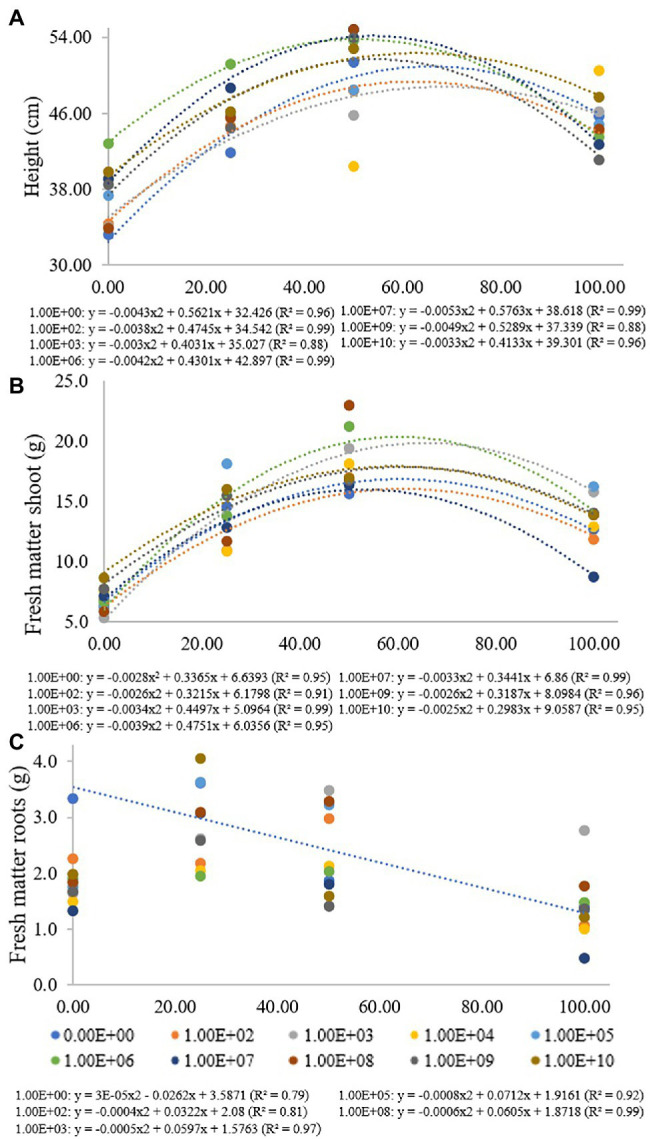
Regression data for the unfolding of the interaction between inoculum concentrations and fertilization doses in relation to **(A)** plant height, **(B)** shoot fresh matter and **(C)** root fresh matter. The X-axis indicates the fertilization dose as a percentage. Colored circles and the equation—0.00E+00 means no inoculation (control); 1.00E+02 means 1 × 10^2^ CFU ml^−1^; 1.00E+03 means 1 × 10^3^ CFU ml^−1^; 1.00E+04 means 1 × 10^4^ CFU ml^−1^; 1.00E+05 means 1 × 10^5^ CFU ml^−1^; 1.00E+06 means 1 × 10^6^ CFU ml^−1^; 1.00E+07 means 1 × 10^7^ CFU ml^−1^; 1.00E+08 means 1 × 10^8^ CFU ml^−1^; 1.00E+09 means 1 × 10^9^ CFU ml^−1^; and 1.00E+10 means 1 × 10^10^ CFU ml^−1^.

For plant height, shoot fresh matter, root fresh matter, CFU of endophytic *B. subtilis* recovered from soybean roots, shoot nitrogen and phosphorus concentration, a significant interaction was observed between inoculum concentration and fertilization dose (*p* < 0.05; Table 1). For plant height, an interaction between inoculum concentration and fertilization dose was observed, and its unfolding showed an adjustment for quadratic regression at inoculum concentrations of 0, 1 × 10^2^, 1 × 10^3^, 1 × 10^6^, 1 × 10^7^, 1 × 10^9^ and 1 × 10^10^ CFU ml^−1^ (*R*^2^: 0.96, 0.99, 0.88, 0.99, 0.99, 0.88 and 0.96, respectively), with maximum points at fertilization doses of 65%, 62%, 67%, 51%, 54%, 53%, and 63%, respectively (Figure 1A).

**Table 1:** Height, shoot fresh matter (SFM), root fresh matter (RFM), CFU of endophytic *Bacillus subtilis* recovered from soybean roots, nitrogen and phosphorus concentrations in soybean plant shoots as a function of fertilization dose.

Value of *p*	Height	SFM	RFM	CFU	*N*	*p*
Inoculation	0.0492	0.0121	0.0087	<0.0001	0.0026	<0.0001
Fertilizer	<0.0001	<0.0001	<0.0001	0.5121	<0.0001	<0.0001
I × F	0.0108	0.0002	0.0015	<0.0001	0.0028	<0.0001
VC (%)	9.33	7.91	17.21	3.80	10.87	11.11
General mean	44.66	44.60	13.01	6.79	38.05	2.06

I, inoculation; F, fertilizer; and VC, variation coefficient.

**Figure 2:**
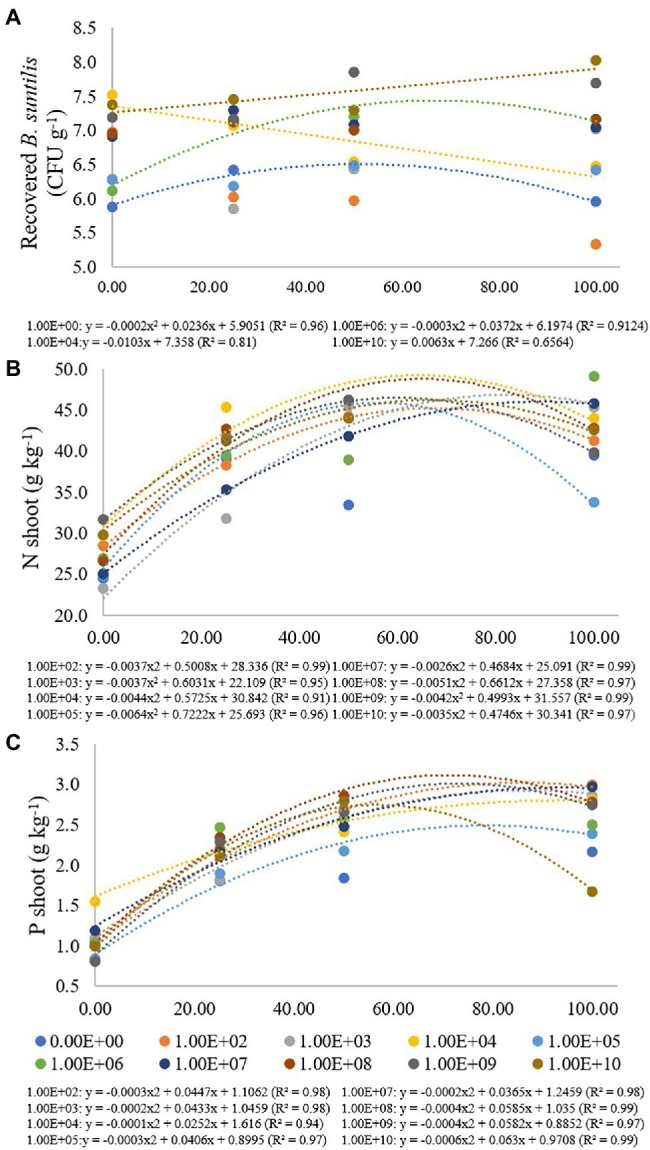
Regression data for the unfolding of the interaction between inoculum concentrations and fertilization doses in relation to the concentration of **(A)** *B. subtilis* recovered from roots (CFU g^−1^); **(B)** nitrogen (g kg^−1^) and **(C)** phosphorus (g kg^−1^) concentration in soybean plant shoots. The X-axis indicates fertilization dose in percentage—colored circles and the equation—0.00E+00 means no inoculation (control); 1.00E+02 means 1 × 10^2^ CFU ml^−1^; 1.00E+03 means 1 × 10^3^ CFU ml^−1^; 1.00E+04 means 1 × 10^4^ CFU ml^−1^; 1.00E+05 means 1 × 10^5^ CFU ml^−1^; 1.00E+06 means 1 × 10^6^ CFU ml^−1^; 1.00E+07 means 1 × 10^7^ CFU ml^−1^; 1.00E+08 means 1 × 10^8^ CFU ml^−1^; 1.00E+09 means 1 × 10^9^ CFU ml^−1^; 1.00E+10 means 1 × 10^10^ CFU ml^−1^.

At a fertilization dose of 0%, the inoculum concentration of 1 × 10^6^ promoted the largest plants with a height of 42.8 cm, and this inoculation resulted in a regression equation with a maximum point at a fertilization dose of 51%, where the height estimated was 53.9 cm. At a fertilization dose of 100%, the concentration of 1 × 10^10^ CFU ml^−1^ promoted plants 47.7 cm in height, and the concentration of 1 × 10^9^ CFU ml^−1^ provided the smallest plants at this fertilization dose, 41.1 cm in height (Figure 1A). Analyzing fertilization doses within each inoculant concentration for shoot fresh matter (Figure 1B), there was adjustment for quadratic regression at inoculum concentrations of 0, 1 × 10^2^, 1 × 10^3^, 1 × 10^6^, 1 × 10^7^, 1 × 10^8^, 1 × 10^9^ and at 1 × 10^10^ CFU ml^−1^ (*R*^2^ de 0.95, 0.91, 0.99, 0.95, 0.99, 0.96 and 0.95, respectively), with maximum points at fertilization doses of 60%, 62%, 66%, 61%, 52%, 61% and 60%, respectively. For concentrations of 1 × 10^4^, 1 × 10^5^ and 1 × 10^8^ CFU ml^−1^, there was no regression adjustment (Figure 2B).

For a fertilization dose of 0%, an inoculum concentration of 1 × 10^3^ CFU ml^−1^ provided the smallest plants (5.29 g), but this inoculation resulted in a regression equation that provided higher shoot dry matter values at fertilization doses above 50%. The inoculum concentration of 1 × 10^6^ CFU ml^−1^ also provided higher fresh matter values at fertilization doses above 25%. A fertilization dose of 50%, for example, promoted a shoot fresh matter value of 21.22 g (Figure 1B).

For root fresh matter, inoculum concentrations of 1 × 10^2^, 1 × 10^3^, 1 × 10^5^ and 1 × 10^9^ CFU ml^−1^ were adjusted for quadratic regression equations (*R*^2^ of 0.81, 0.97, 0.92 and 0.99, respectively) with maximum points at fertilization doses of 40%, 60%, 44% and 50%, respectively. Inoculation with 1 × 10^3^ CFU ml^−1^ provided greater root fresh matter at a fertilization dose of 100% (2.76 g). Treatment without bacterial inoculation was adjusted in a decreasing linear regression (*R*^2^: 0.79), with 3.33 and 1.39 g of roots at fertilization doses of 0% and 100%, respectively. For concentrations of 1 × 10^7^ and 1 × 10^10^ CFU ml^−1^, there was no regression adjustment, and for concentrations of 1 × 10^4^, 1 × 10^6^ and 1 × 10^9^ CFU ml^−1^, there were no significant differences for the fertilization doses used (Figure 1C).

In the treatment where bacterial inoculation was not performed, the lowest *B. subtilis* concentration recovered from roots was verified at a fertilization dose of 0%, with adjustment for quadratic regression (*R*^2^ of 0.96), resulting in a maximum point at a fertilization dose of 59%. For a concentration of 1 × 10^6^ CFU ml^−1^, there was also an adjustment for quadratic regression (*R*^2^ of 0.91), and the maximum point was obtained at a fertilization dose of 62% (Figure 2A).

**Figure 3:**
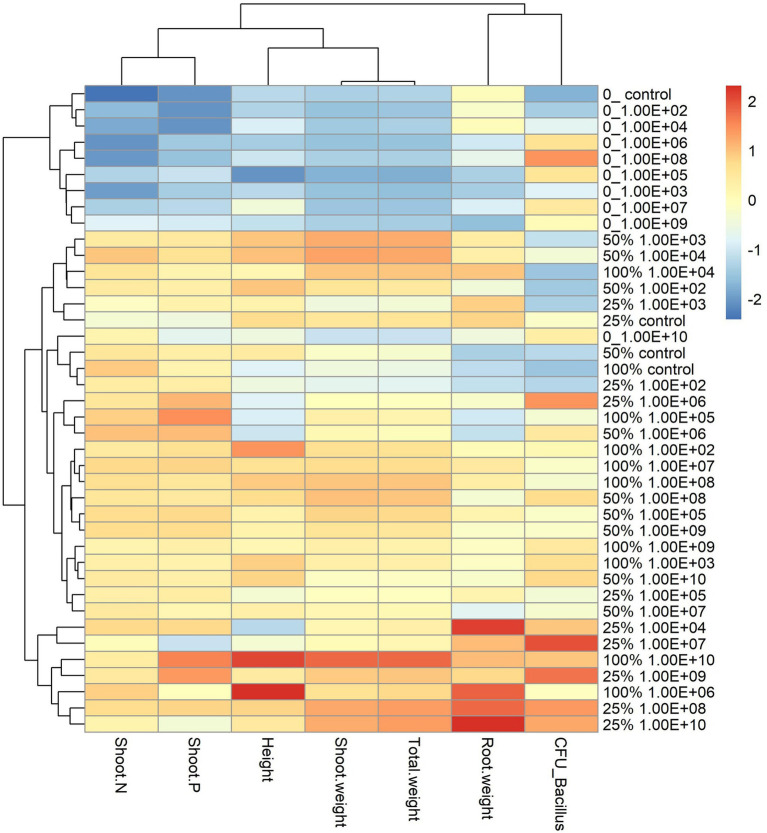
Heatmap with cluster analysis. The column corresponds to inoculation concentrations and mineral fertilization doses and the line to the biometric parameters of the soybean plant. The Y-axis—0_ indicates the fertilization dose, and 1.00E+02 indicates the inoculum concentration. The fertilization doses were 0%, 25%, 50% and 100%, and the fertilization doses were 1 × 10^2^ CFU ml^−1^, 1 × 10^3^ CFU ml^−1^, 1 × 10^4^ CFU ml^−1^, 1 × 10^5^ CFU ml^−1^, 1 × 10^6^ CFU ml^−1^, 1 × 10^7^ CFU ml^−1^, 1 × 10^8^ CFU ml^−1^, 1 × 10^9^ CFU ml^−1^, and 1 × 10^10^ CFU ml^−1^, representing 1.00E+02, 1.00E+03, 1.00E+04, 1.00E+05, 1.00E+06, 1.00E+07, 1.00E+08, 1.00E+09, and 1.00E+10, respectively. The X-axis indicates the parameters of plant growth—total weight (g); shoot weight (g); height (cm); shoot P—phosphorus content in shoot g kg^−1^; shoot N—nitrogen content in shoot g kg^−1^; CFU_ Bacillus—amount of *Bacillus* reisolated from root CFU g^−1^ and root weight (g).

For an inoculum concentration of 1 × 10^4^ CFU ml^−1^, a fertilization dose of 0% provided the highest recovered *B. subtilis* concentration, and while the fertilization doses increased, the number of recovered *B. subtilis* decreased. An inoculum concentration of 1 × 10^10^ CFU ml^−1^ also promoted a high number of recovered *B. subtilis* at a fertilization dose of 0%. The values were adjusted in decreasing (*R*^2^: 0.81) and increasing (*R*^2^: 0.66) linear regressions. For inoculum concentrations of 1 × 10^2^, 1 × 10^3^ and 1 × 10^9^ CFU ml^−1^, there was no regression adjustment, and for concentrations of 1 × 10^5^, 1 × 10^7^ and 1 × 10^8^ CFU ml^−1^, there were no significant differences for the fertilization doses used (Figure 3A).

For shoot nitrogen content, inoculum concentrations of 1 × 10^2^, 1 × 10^3^, 1 × 10^4^, 1 × 10^5^, 1 × 10^7^, 1 × 10^8^, 1 × 10^9^ and 1 × 10^10^ CFU ml^−1^ had adjustment for quadratic regression equations (*R*^2^ of 0.99, 0.95, 0.91, 0.96, 0.99, 0.97, 0.99 and 0.97, respectively) with maximum points at fertilization doses of 68%, 81%, 65%, 56%, 90%, 65%, 59% and 68%, respectively. For the treatment that received no inoculation and for the inoculum concentration of 1 × 10^6^ CFU ml^−1^, there was no regression adjustment (Figure 2B). Concentrations of 1 × 10^3^ and 1 × 10^7^ CFU ml^−1^ resulted in the lowest N contents when there was no fertilizer application, but at the maximum fertilization dose, these inoculations resulted in higher shoot N contents, 45.35 and 45.78 g kg^−1^, respectively (Figure 2B).

For the shoot phosphorus concentration, inoculum concentrations of 1 × 10^2^, 1 × 10^3^, 1 × 10^4^, 1 × 10^5^, 1 × 10^7^, 1 × 10^8^, 1 × 10^9^ and 1 × 10^10^ CFU were adjusted for quadratic regression equations (*R*^2^ of 0.98, 0.98, 0.914, 0.97, 0.98, 0.99, 0.97 and 0.99, respectively), with maximum points at fertilization doses of 74%, 108%, 126%, 68%, 91%, 73%, 72% and 52%, respectively (Figure 2C). For treatment that did not receive inoculation and for an inoculum concentration of 1 × 10^6^ CFU mL^−1^, there was no regression adjustment. Phosphorus concentrations ranged from 0.81 and 2.99 g kg^−1^ at concentrations of 1 × 10^3^ and 1 × 10^6^ CFU ml^−1^ and fertilization doses of 0% and 100%, respectively (Figure 3C).

### Maize Crop

**Figure 4:**
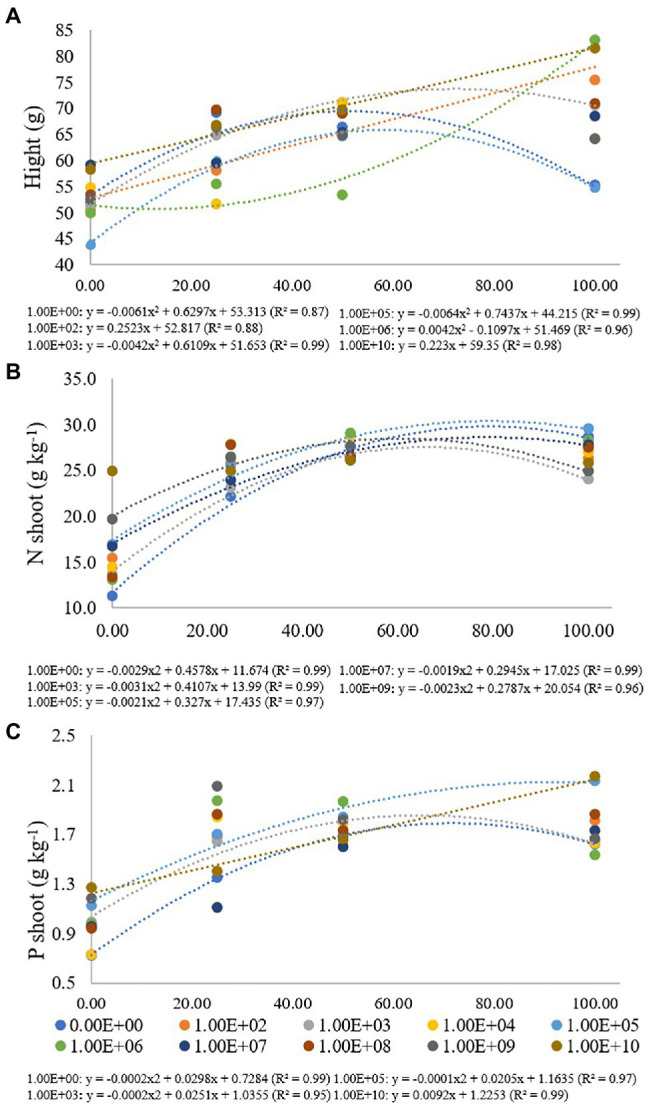
Regression data for the unfolding of the interaction between inoculum concentrations and fertilization doses in relation to **(A)** height (cm) of maize plants; **(B)** nitrogen (g kg^−1^) and **(C)** phosphorus (g kg^−1^) concentration in maize plant shoots. The X-axis indicates fertilization dose in percentage—colored circles and the equation—0.00E+00 means no inoculation (control); 1.00E+02 means 1 × 10^2^ CFU ml^−1^; 1.00E+03 means 1 × 10^3^ CFU ml^−1^; 1.00E+04 means 1 × 10^4^ CFU ml^−1^; 1.00E+05 means 1 × 10^5^ CFU ml^−1^; 1.00E+06 means 1 × 10^6^ CFU ml^−1^; 1.00E+07 means 1 × 10^7^ CFU ml^−1^; 1.00E+08 means 1 × 10^8^ CFU ml^−1^; 1.00E+09 means 1 × 10^9^ CFU ml^−1^; 1.00E+10 means 1 × 10^10^ CFU ml^−1^.

For plant height and nitrogen and phosphorus concentrations in maize plant shoots, a significant interaction was verified between inoculum concentration and fertilization dose (*p* < 0.05; Table 2), and its unfolding was performed (Figure 4).

**Table 2:** Height, SFM, RFM, CFU of endophytic *Bacillus subtilis* recovered from soybean roots, and nitrogen and phosphorus concentrations in maize plant shoots as a function of fertilization dose.

Value of *p*	Height	SFM	RFM	CFU	*N*	*P*
Inoculation	0.0051	0.1323	0.0648	<0.0001	<0.0001	<0.0001
Fertilizer	<0.0001	<0.0001	0.0002	0.0029	<0.0001	<0.0001
I × F	0.0017	0.3064	0.2533	0.0202	<0.0001	<0.0001
VC (%)	11.68	33.43	43.80	7.74	6.07	8.73
General mean	62.60	37.17	4.27	7.24	23.89	1.54

I, inoculation; F, fertilizer; and VC, variation coefficient.

For the height of maize plants, treatment without inoculation and inoculum concentrations of 1 × 10^3^, 1 × 10^5^ and 1 × 10^6^ CFU ml^−1^ were adjusted for quadratic regression equations (*R*^2^ of 0.87, 0.99, 0.99 and 0.96, respectively). For treatments without inoculation and concentrations of 1 × 10^3^ and 1 × 10^5^ CFU ml^−1^, the maximum point was verified at fertilization doses of 51%, 72% and 58%, with heights from 43.83 to 83.17 cm. For a concentration of 1 × 10^6^ CFU ml^−1^, there was no adjustment for adjusted regression. Inoculum concentrations of 1 × 10^2^ and 1 × 10^10^ CFU ml^−1^ were adjusted for a regression (*R*^2^: 0.88 and 0.98) with a progressive increase in plant height according to the high fertilization doses, with heights of 75.42 for 1 × 10^2^ and 81.50 cm for 1 × 10^10^ CFU ml^−1^ at a fertilization dose of 100%. For inoculum concentrations of 1 × 10^4^ and 1 × 10^8^, there was no regression adjustment, and for concentrations 1 × 10^7^ and 1 × 10^9^ CFU ml^−1^, there were no significant differences for fertilization doses used (Figure 4A).

For the nitrogen concentration in maize plants, treatment without inoculation and inoculum concentrations of 1 × 10^3^, 1 × 10^5^ and 1 × 10^7^ and 1 × 10^9^ CFU ml^−1^ were adjusted for quadratic regression equations (*R*^2^ of 0.99, 0.99, 0.97, 0.99 and 0.96, respectively) with maximum points at fertilization doses of 79%, 66%, 78%, 77% and 61%, respectively, with nitrogen concentrations ranging from 11.33 to 29.64 g kg^−1^. There was no regression adjustment for concentrations of 1 × 10^2^, 1 × 10^4^, 1 × 10^6^, 1 × 10^8^ and 1 × 10^9^ CFU ml^−1^, and for a concentration of 1 × 10^10^ CFU ml^−1^, there was no significant difference for fertilization doses used (Figure 4B).

Phosphorus values in maize plants ranged from 0.73 to 2.17 g kg^−1^, and treatment without inoculation and inoculum concentrations of 1 × 10^3^ and 1 × 10^5^ CFU ml^−1^ were adjusted for quadratic regression equations (*R*^2^ of 0.99, 0.95 and 0.97, respectively) with maximum points at fertilization doses of 74%, 63% and 102%, respectively. A concentration of 1 × 10^10^ CFU ml^−1^ was adjusted in an increasing linear regression with values of 1.27, 1.41, 1.67 and 2.17 g kg^−1^ for fertilization doses of 0%, 25%, 50% and 100%, respectively. There was no regression adjustment for concentrations of 1 × 10^2^, 1 × 10^4^, 1 × 10^6^, 1 × 10^7^, 1 × 10^8^ and 1 × 10^9^ CFU ml^−1^ (Figure 4C).

The analysis of the heatmap graph with cluster analysis for soybean plants showed that treatments that did not receive fertilization had the lowest values for all analyzed parameters and that even the highest inoculant concentrations did not offset the lack of fertilization (Figure 3). On the other hand, treatments that received a mineral fertilizer dose of 50% regardless of inoculant concentrations had the highest values for parameters related to plant development.

**Figure 5:**
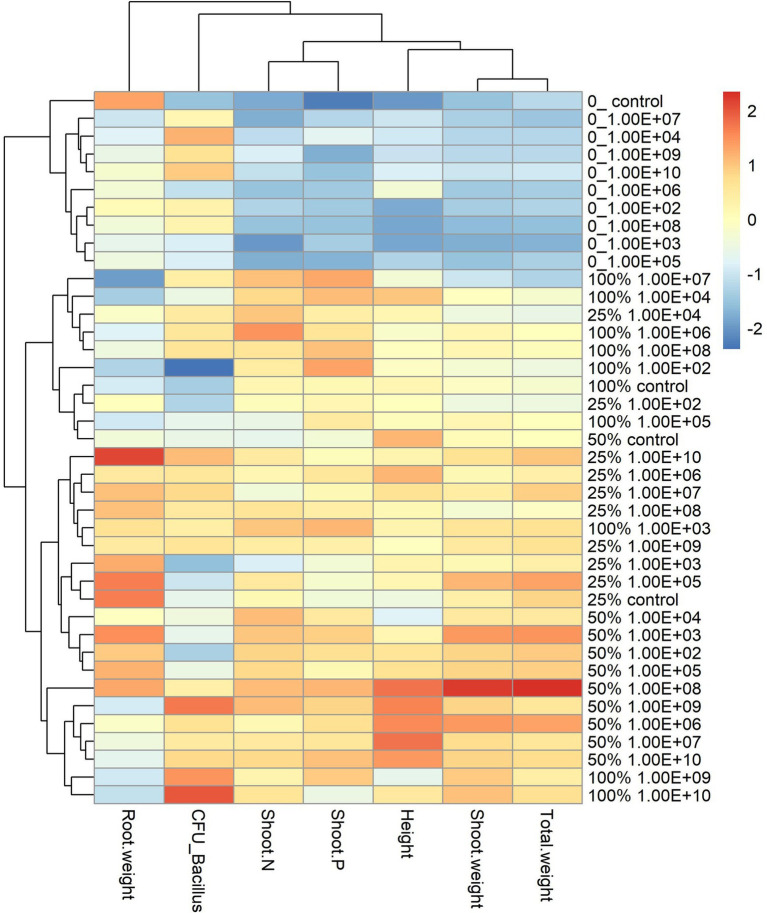
Heatmap with cluster analysis. The column corresponds to inoculation concentrations and mineral fertilization doses and the line to the biometric parameters of the maize plant. The Y axis—0_ indicates the fertilization dose, and 1.00E+02 indicates the inoculum concentration. The fertilization doses were 0%, 25%, 50% and 100%, and the fertilization doses were 1 × 10^2^ CFU ml^−1^, 1 × 10^3^ CFU ml^−1^, 1 × 10^4^ CFU ml^−1^, 1 × 10^5^ CFU ml^−1^, 1 × 10^6^ CFU ml^−1^, 1 × 10^7^ CFU ml^−1^, 1 × 10^8^ CFU ml^−1^, 1 × 10^9^ CFU ml^−1^, and 1 × 10^10^ CFU ml^−1^, representing 1.00E+02, 1.00E+03, 1.00E+04, 1.00E+05, 1.00E+06, 1.00E+07, 1.00E+08, 1.00E+09, and 1.00E+10, respectively. The X-axis indicates the parameters of plant growth—total weight (g); shoot weight (g); height (cm); shoot P—phosphorus content in shoot g kg^−1^; shoot N—nitrogen content in shoot g kg^−1^; CFU_ Bacillus—amount of *Bacillus* reisolated from root CFU g^−1^ and root weight (g).

For the maize plant, the heatmap graph showed values similar to soybean plants in the absence of fertilization; however, parameters related to plant development were higher for fertilization doses of 25% and 50%. For a fertilization dose of 25%, the best concentrations according to the heatmap analysis were 1 × 10^8^ and 1 × 10^10^ CFU ml^−1^ (Figure 5).

## Discussion

For soybean plants, at inoculum concentrations of 1 × 10^6^ CFU ml^−1^ and 1 × 10^7^ CFU ml^−1^, the results showed the highest plant height values for the lowest fertilization doses, 51% and 54% of the recommended dose. When the inoculant concentration was increased to 1 × 10^10^ CFU ml^−1^, to reach the same height value, the need for fertilization increased to 63% of the recommended inoculant dose.

For shoot fresh matter, inoculant concentrations of 1 × 10^7^ CFU ml^−1^ promoted the highest SFM value, requiring only a fertilization dose of 52%. When the inoculant concentration was 1 × 10^10^ CFU ml^−1^, the fertilization dose needed to be increased to 62% of the recommended dose, maintaining the SFM value.

For root fresh matter, the highest value was found at an inoculant concentration of 1 × 10^5^ CFU ml^−1^ with the need for a fertilization dose of 44%, while when the inoculant concentration was 1 × 10^9^ CFU ml^−1^, the fertilizer requirement increased to 50% of the recommended dose for the maximum RFM value.

The results show that there is a strong tendency for the inoculant concentration to increase, and the need for mineral fertilization dose is consequently increased to achieve the same plant development.

The highest number of *B. subtilis* recovered from root soybean was found at inoculum concentrations of 1 × 10^4^ CFU ml^−1^ and 1 × 10^10^ ml^−1^ with no mineral fertilization (Figure 3A). When mineral fertilization occurred, the inoculum concentration did not alter the number of *B. subtilis* recovered. These results suggest that mineral fertilization interferes with the number of endophytes regardless of the bacterial inoculum concentration. On the other hand, the highest bacterial inoculum concentrations did not promote the highest plant growth. Mineral fertilization limited the colonization of *B. subtilis* in the root. Most likely, it did not limit the colonization of *B. subtilis* in the rhizosphere. Unfortunately, it was not measured in this study. It is important to note that the microbial population in the rhizospheric soil is approximately 1 × 10^2^–1 × 10^6^ CFU g^−1^ (Nihorimbere et al., 2011; Huang et al., 2014). The results showed that the highest inoculum concentrations of 1 × 10^9^ and 1 × 10^10^ CFU ml^−1^ increased the need for soil fertility by the plants. We proposed that these high inoculum concentrations promoted an increase in the bacterial rhizospheric population, and as a consequence, more photosynthetic metabolites from the plants and more nutrients from the soil are needed. Thus, the need for mineral fertilization for plant growth must be increased.

*Bacillus subtilis* used in this study has several abilities to promote plant growth, such as the synthesis of phytohormones, biological nitrogen fixation, promotion of root and shoot development and increased productivity in several crops, such as soybean, maize, cotton and sugarcane (dos Santos et al., 2018; Lobo et al., 2019; Shastri et al., 2020; Dos Santos et al., 2021; Escobar Diaz et al., 2021). However, in previous studies, the *B. subtilis* concentration used was 1 × 10^9^ CFU ml^−1^, and the fertilization dose was 100% the recommended dose for each crop.

Nascimento et al. (2020) evaluated several bacterial inoculants, including *Bacillus pumilus* and *B. amyloliquefaciens,* in the presence and absence of mineral fertilization and measured several parameters related to plant development. The results of this study showed that *Bacillus* inoculation, even in the absence of mineral fertilization, increased phosphorus concentrations in plant roots and shoots compared to fertilized plants.

Agbodjato et al. (2021) assessed the efficacy of solid biostimulants formulated from the rhizobacteria *Pseudomonas putida* and different binders on maize cultivation in the farming environment. The results obtained show that the best height, stem diameter, and leaf area were obtained by applying biostimulants based on *P. putida* and half doses of NPK and urea.

Microbial-based biofertilizers are among the key agricultural components that improve crop productivity and contribute to sustainable agroecosystems (Souza et al., 2015; Bargaz et al., 2018; Aziz et al., 2019; Tosi et al., 2021). The amount of mineral fertilizer for crop production has increased annually. However, the average recovery efficiencies range from 20% to 50%, depending on the nutrient (Sarkar et al., 2021). Some recent studies have shown improved nutrient use efficiency due to microbial inoculation of plants (Adesemoye and Kloepper, 2009; Abo-Kora, 2016; Paungfoo-Lonhienne et al., 2019; Pereira et al., 2020; Sarkar et al., 2021). However, these studies did not evaluate the effect of high inoculum concentrations on plant growth. Escobar Diaz et al. (2021) assessed the impact of different inoculum concentrations, including *B. subtilis*, on cotton crops and did not find a difference between yield crops when the plants received the inoculum at 1 × 10^4^ and 1 × 10^10^ CFU ml^−1^. However, these authors evaluated the effect of different inoculum concentrations at 100% of the fertilization dose, only. The present study associated the bacterial inoculant concentration with mineral fertilization doses and the effect of these two parameters on the colonization of the endophytic bacterium *B. subtilis*.

The first step for the effect of plant growth promotion by bacterial inoculants to occur is the need for rhizosphere colonization and subsequent plant tissue colonization. Modulation of the rhizosphere microbiota is affected by several factors, such as plant genotype, development stage, soil properties, climatic conditions, agricultural practices and other biotic and abiotic factors (Balsanelli et al., 2010; Compant et al., 2019).

Inoculum bacteria can transfer their abilities to bacteria in the natural bacterial community. This process is called HGT—horizontal gene transfer, which is a mechanism for transferring genetic information between phylogenetically distant microbial species, providing microorganisms with new functions that facilitate their adaptation to the environment (Tiwari and Bae, 2020).

In addition, inoculant bacteria can communicate, coordinate and synchronize the behavior of natural microbial communities, such as aggregation, virulence, biofilm formation and secondary metabolic production, through quorum sensing. Quorum sensing is a regulatory mechanism based on the secretion of signaling molecules that can be detected by some members of the bacterial community, promoting specific responses in the receiving bacteria (Sharma et al., 2020).

Another example of a well-studied adaptation that increases bacterial competitiveness in densely populated habitats such as the rhizosphere is the synthesis of type VI secretion systems (T6SS), which gives bacteria the ability to introduce toxic proteins into neighboring cells (Bernal et al., 2018). Thus, the production of molecules with antimicrobial activity, resistance to antimicrobial molecules produced by other organisms, evasion of predatory organisms and ability to metabolize different compounds make endophytic bacteria great competitors, which is essential for plant colonization. Through these mechanisms, synergistic cooperation, competition and antagonistic interactions occur within the rhizosphere, creating networks that structure bacterial communities (Hassani et al., 2018; Levy et al., 2018).

These studies show that the individual abilities of bacterial species present in the inoculant are certainly more effective in terms of colonization and, consequently, of plant growth promotion compared to the bacterial inoculum concentration, which does not mean that the latter is unimportant.

Inoculant concentrations of 1 × 10^4^ and 1 × 10^10^ CFU ml^−1^ promoted the highest number of endophytic *Bacillus* recovered from roots. Both treatments did not receive mineral fertilization (fertilization dose of 0%). When mineral fertilization was applied, the number of recovered endophytic *Bacillus* decreased.

The use of chemical fertilizers in agricultural production provides an average increase in productivity of approximately 50% in relation to production without their use; however, chemical fertilization practices disregard the biological potential of roots or rhizosphere, increasing the mobilization and acquisition of nutrients and decreasing interactions between plants and rhizospheric microorganisms (Meena et al., 2017). Most likely, the decrease in the number of *Bacillus* recovered (endophytics) from roots with mineral fertilization is a consequence of the plant’s lower need for bacteria when there is greater availability of nutrients.

For maize plants, the tendency of higher inoculant concentrations to increase the need for mineral fertilization was lower than for soybean plants. Regarding height, an inoculant concentration of 1 × 10^5^ CFU ml^−1^ promoted the need for only 58% of the recommended fertilization dose, while a lower concentration of 1 × 10^3^ CFU ml^−1^ increased the fertilization requirement to 72% of the recommended dose.

For the nitrogen concentration in maize plants, an inoculum concentration of 1 × 10^3^ CFU ml^−1^ promoted the need for only 66% of the recommended fertilization dose. The concentration of 1 × 10^9^ CFU ml^−1^ required only a fertilization dose of 61%, and the maximum phosphorus concentration was reached at an inoculant concentration of 1 × 10^3^ CFU ml^−1^, requiring 63% of the recommended fertilization dose.

The means by which PGPR improve the nutritional status of host plants can be categorized into five areas: (1) biological nitrogen fixation, (2) phosphorus solubilization by increasing the availability of this nutrient in the rhizosphere, (3) inducing increases in the root surface area, (4) increasing other beneficial host symbiosis, and (5) a combination of previous modes of action (Da Fonseca Breda et al., 2019). In fact, the set of these characteristics is more important than just the concentration of the inoculum.

## Conclusion

The results show different responses regarding inoculum concentrations and fertilization doses for soybean and maize crops. There is a strong tendency for soybean plants with the increase in the *B. subtilis* inoculant concentration and the need for mineral fertilization doses to achieve the same plant development. The findings suggest, for soybean crops, that these high inoculum concentrations required more photosynthetic metabolites from the plants and more nutrients from the soil. Thus, the need for mineral fertilization for plant growth must be increased. For maize plants, there was no such tendency. The findings have shown that fertilization dose and inoculum concentration interfere with plant growth differently depending on the crop.

## Data Availability Statement

The datasets presented in this study can be found in online repositories. The names of the repository/repositories and accession number(s) can be found in the article/Supplementary Material.

## Author Contributions

All authors listed have made a substantial, direct, and intellectual contribution to the work and approved it for publication.

## Funding

This work was carried out with support from the Coordination of Improvement of Higher Education Personnel—Brazil (CAPES)—Financing Code 001.

## Conflict of Interest

The authors declare that the research was conducted in the absence of any commercial or financial relationships that could be construed as a potential conflict of interest.

## Publisher’s Note

All claims expressed in this article are solely those of the authors and do not necessarily represent those of their affiliated organizations, or those of the publisher, the editors and the reviewers. Any product that may be evaluated in this article, or claim that may be made by its manufacturer, is not guaranteed or endorsed by the publisher.
