# Supplementary material for: Effects of Chemical Fertilization and Microbial Inoculum on Bacillus subtilis Colonization in Soybean and Maize Plants
Source: Front Microbiol. 2022 Jul 6;13:901157. doi: 10.3389/fmicb.2022.901157 (PMC9298503; doi:10.3389/fmicb.2022.901157)
Supplement: Supplementary file 1 [file Image_1.pdf]

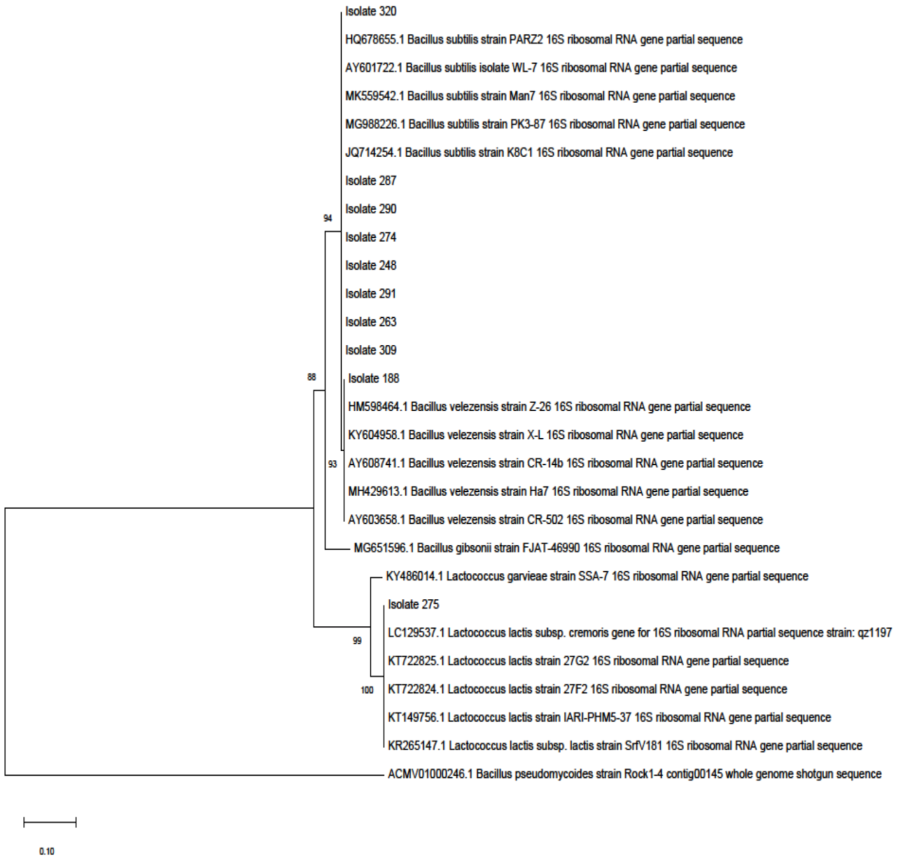


**S1 -** Phylogenetic tree based on the nucleotide sequence of microorganisms obtained from the NCBI database (number of indicated accession), showing the phylogenetic relationship with the isolates *Bacillus subtilis* 290.
